# From Inflammation to Malignancy: The Link Between Endometriosis and Gynecological Cancers

**DOI:** 10.3390/ijms262411816

**Published:** 2025-12-07

**Authors:** Karolina Kłodnicka, Aleksandra Michalska, Jacek Januszewski, Alicja Forma, Grzegorz Teresiński, Jolanta Flieger, Jacek Bogucki, Marcin Maciejewski, Kinga Syty, Jacek Baj

**Affiliations:** 1 Department of Forensic Medicine, Medical University of Lublin, Jaczewskiego 8b, 20-090 Lublin, Poland; 2 Department of Correct, Clinical, and Imaging Anatomy, Medical University of Lublin, Jaczewskiego 4, 20-090 Lublin, Poland; 3 Doctoral School, Medical University of Lublin, Chodźki 7, 20-093 Lublin, Poland; 4 Department of Analytical Chemistry, Medical University of Lublin, Chodźki 4a (Collegium Pharmaceuticum), 20-093 Lublin, Poland; jolanta.flieger@umlub.edu.pl; 5 Institute of Medical Sciences, The John Paul II Catholic University of Lublin, Konstantynów 1, 20-708 Lublin, Polandkinga.syty@kul.pl (K.S.)

**Keywords:** endometriosis, gynecological cancer, endometrial cancer, cervical cancer, ovarian cancer

## Abstract

Endometriosis, a chronic estrogen-dependent disease, is associated with a risk of developing gynecological cancers. The mechanisms of this association remain unclear, but emerging evidence implicates key signaling pathways, including PI3K/AKT/mTOR and *ARID1A* alterations, in malignant transformation. This article examines current reports on the association between endometriosis and cervical, ovarian, and endometrial cancers, with particular emphasis on diagnostic challenges and molecular mechanisms. Imaging methods such as ultrasound, magnetic resonance imaging, and computed tomography (CT) are used for diagnosis, as well as biomarkers such as Cancer Antigen-125 (CA-125) and Human Epididymal protein 4 (HE4), but their specificity is limited, motivating research into novel molecular and non-invasive diagnostics. Laparoscopy is an invasive diagnostic method, serving as the gold standard for confirming the diagnosis. We discuss personalized clinical strategies, including risk-based surveillance for patients with atypical lesions or *ARID1A* alterations, and implications for ovarian cancer management in endometriosis. Prospective cohort studies will be necessary to further understand the complex mechanisms of endometriosis’s malignant transformation. Optimizing therapy and improving quality of life require a holistic, individualized approach to patient care. This review provides an integrated synthesis of epidemiological and molecular evidence, highlighting both established and emerging targets for diagnosis and treatment in endometriosis-associated malignancies.

## 1. Introduction

### 1.1. Definition and Prevalence of Endometriosis

**Figure 1:**
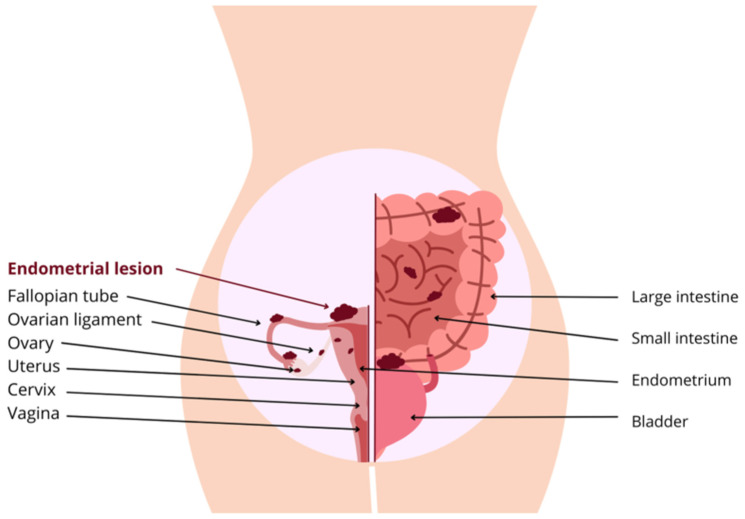
Common abdominal and pelvic sites of endometriotic lesions.

Endometriosis is a chronic inflammatory disease characterized by the presence of endometrial-like tissue outside the uterine cavity [1]. Ectopic lesions most frequently involve the ovaries and peritoneal surfaces of the pelvis but may also be found on the uterine serosa, rectovaginal septum, and, less commonly, on the diaphragm [2]. Endometrial lesions have additionally been reported in the lungs, liver, pericardium, surgical scars, limbs, and even within the central nervous system [3]. However, the true prevalence is likely underestimated because of diagnostic delay, heterogeneity of symptoms, and the frequent absence of pathognomonic features [4]. Although some individuals remain asymptomatic, many present with a broad spectrum of clinical manifestations [5]. Although endometriosis may be asymptomatic, affected individuals often present with a range of symptoms [6]. The disease is classically associated with chronic pelvic pain and infertility, while additional manifestations may include fatigue, gastrointestinal (GI), and urinary symptoms [7]. Approximately 30% of patients with endometriosis develop chronic pelvic pain refractory to standard medical therapies [8]. Infertility occurs in about one-third of women with endometriosis, a prevalence nearly twice that observed in women without the condition [9]. Patients may also present with atypical symptoms, including subfebrile states, nausea, dizziness, headaches, depressive symptoms, anxiety, and hypoglycemia. In some cases, rectal bleeding, hematuria during menstruation, and increased susceptibility to infections and allergies have been reported [10]. The chronic nature of symptoms, particularly pelvic pain and subfertility, may substantially impair social functioning, professional and academic performance, and overall quality of life, especially in young women [11]. Endometriosis encompasses diverse clinical and anatomical presentations and is commonly divided into three major forms [12]: ovarian endometrioma (OMA), superficial peritoneal endometriosis (SUP), and deep infiltrating endometriosis (DIE) [13]. OMAs are benign, cystic ovarian lesions lined by endometrial-type epithelium and stroma that undergo cyclic hemorrhage [14]. This form is the most frequent subtype of endometriosis, affecting between 17% and 44% of women diagnosed with the disease worldwide [15]. Large endometriomas may reduce the follicular reserve by exerting pressure on the ovarian cortex and by inducing proinflammatory immune mediators [16]. SUP is the mildest form of the disease, characterized by endometrial lesions on the peritoneum (the tissue that lines the pelvic cavity) [17]. The most severe form of endometriosis is DIE [18]. It is defined as the presence of lesions that penetrate more than 5 mm beneath the peritoneal surface [19]. It typically presents as nodular lesions capable of invading adjacent structures and is associated with fibrosis and disruption of normal anatomy (Figure 1) [20].

### 1.2. Overview of Gynecological Cancers

Gynecological malignancies constitute a heterogeneous group of tumors arising from the female reproductive tract, including the endometrium, ovary, cervix, vulva, and vagina. Among these, cancers of the uterus (predominantly endometrial carcinoma), cervix, and ovary account for the vast majority of cases worldwide [21]. Collectively, gynecologic cancers affect more than one million women annually, with approximately 500,000 deaths attributed to these malignancies each year [22]. Epithelial ovarian cancer (EOC) represents the leading cause of death from gynecological malignancies because it is often diagnosed at an advanced stage, owing to its deep pelvic location and the paucity of specific early symptoms [23,24]. Endometrial cancer (EC) is the most prevalent gynecological malignancy in high-income countries [25]. In approximately 80% of cases, disease is confined to the uterus at diagnosis and presents with abnormal uterine bleeding, typically postmenopausal, which facilitates earlier detection and generally favorable prognosis [26]. Cervical cancer remains one of the most prevalent cancers in women worldwide, ranking fourth after breast, colorectal, and lung cancers [27]. It disproportionately affects low- and middle-income countries, which account for more than 85% of cases globally [28]. Although early-stage disease is often asymptomatic, it can be identified through effective screening methods such as Pap smears testing [29]. Human papillomavirus (HPV) infection is a well-established cause of cervical cancer in women [30]. While vaccination has reduced the prevalence of the most common high-risk HPV serotypes (HPV16 and HPV18), both the incidence and mortality of cervical cancer remain high [31]. In summary, although gynecological cancers share certain anatomical and hormonal contexts, their etiologies are highly heterogeneous. The clearest and most consistent evidence for an association with endometriosis concerns endometrioid and clear cell ovarian carcinomas. Endometrial carcinoma may share overlapping molecular pathways, whereas cervical carcinoma exemplifies a malignancy without an etiological connection to endometriosis. This distinction underscores the importance of analyzing both associated and non-associated cancers to better delineate the specific oncogenic mechanisms linked to endometriosis.

### 1.3. Epidemiological Evidence and Link Between Endometriosis and Increased Cancer Risk

For many years, the association between endometriosis and malignancy has been the focus of extensive scientific investigation [32]. Accumulating evidence suggests that certain gynecological cancers may originate from endometriosis, a hypothesis reinforced by findings from the past few decades [33]. The malignant transformation of endometriosis has been linked to factors such as hormonal dysregulation, reproductive history, environmental exposures, and underlying genetic susceptibility [34]. Breast cancer, along with endometriosis, is an estrogen-dependent condition that shares risk factors related to reproductive history and hormone replacement therapy (HRT) [35]. Evidence from large cohort and case–control studies shows an elevated risk of endometrial and breast cancer among women with endometriosis [36]. In addition, it has been reported that patients with endometriosis are at increased risk of developing uterine sarcoma [37]. The strongest epidemiological evidence, however, concerns ovarian cancer. There is growing evidence supporting an association between endometriosis and a higher risk of this malignancy [38]. Ovarian epithelial tumors may originate within endometriosis or from cortical inclusions of Müllerian epithelium, which are likely a form of endosalpingiosis [39]. Women affected by the disease have a 4.2-fold higher risk compared to those without endometriosis [40]. Some studies suggest that women with endometriosis may have a decreased risk of cervical cancer of squamous cell histology [41]. Nevertheless, endometriosis has also been linked to an elevated mortality risk from gynecological cancers [42]. These findings indicate that the carcinogenic potential of endometriosis is largely restricted to tumors arising from endometrioid or clear cell lineages of the ovary. This reflects their shared molecular alterations and the chronic inflammatory microenvironment that facilitates malignant transformation.

### 1.4. Rationale and Objective of the Review

Although endometriosis has long been recognized as a benign gynecological disorder, accumulating evidence suggests that it may influence the development of several malignancies. Most attention has been directed toward its association with ovarian cancer, but potential links with endometrial, breast, and other cancers have also been reported. Given the high prevalence of endometriosis and the clinical significance of cancer risk, a comprehensive synthesis of current evidence is warranted. This review aims to provide an integrated comparison of the molecular and clinical relationships between endometriosis and various gynecologic malignancies, emphasizing cancers in which a causal link has been established as well as those in which no etiological association exists (e.g., cervical carcinoma). This comparative approach facilitates the identification of shared and divergent pathogenic pathways, offering insights into cancer-specific risk modulation in women with endometriosis.

## 2. Pathophysiology of Endometriosis

**Figure 2:**
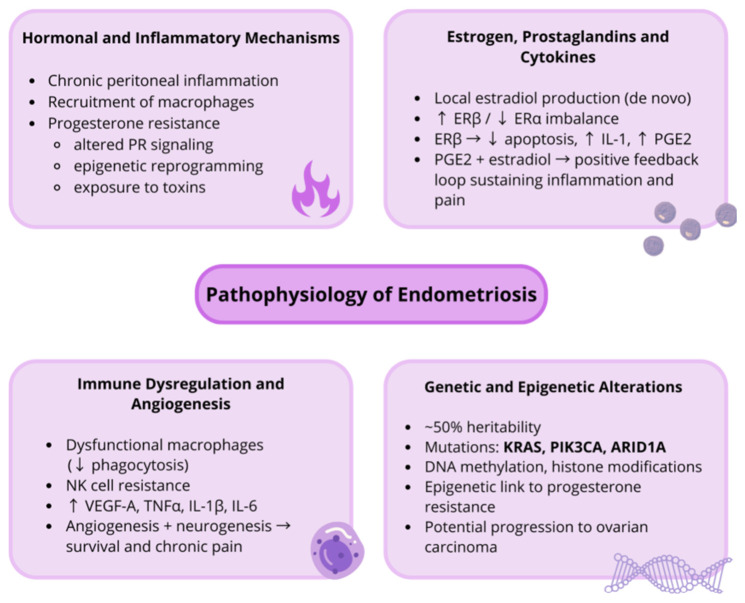
Pathophysiology of Endometriosis including hormonal and inflammatory mechanisms, signaling molecules role, immune dysregulation and angiogenesis and genetic abnormalities.

Endometriosis is a chronicestrogen-dependent inflammatory gynecological disorder characterized by complex interactions among hormonal, immune, and genetic factors. Understanding its pathogenesis requires an appreciation of the physiological structure and function of the uterus and its endometrial lining. The uterus, a mesodermal derivative, is a hollow, thick-walled, muscular organ with distinctive anatomical and histological properties [43]. The endometrium, which forms the uterine lining, is a complex tissue composed of a single epithelial layer overlying a mesenchymal stroma [44]. Functionally, the endometrium prepares for implantation, supports early pregnancy, and undergoes cyclical shedding during menstruation in the absence of fertilization [45]. Throughout a woman’s lifespan, the endometrium undergoes repeated cycles of regeneration as part of its normal physiology [46]. Among differentiated human tissues, it exhibits one of the highest proliferation rates [47] with its growth and differentiation tightly regulated by ovarian hormones, particularly estrogen [48]. Reproductive and hormonal factors, including early menarche, shorter menstrual cycles, and low body mass index, have been associated with an elevated risk of endometriosis [49]. Repeated exposure to ovulatory cycles appears to be the strongest risk factor [50]. These observations highlight the central role of cyclic hormonal activity in the pathogenesis of endometriosis. The disease develops through the interplay of endocrine, immune, and genetic mechanisms, reflecting its multifactorial and systemic nature [51] (Figure 2).

### 2.1. Theories on the Origin of Endometriosis

The hypothesized sources of endometriotic tissue involve retrograde menstruation, coelomic metaplasia, and lymphatic and vascular metastasis [52]. The hypothesis that endometriosis may develop from coelomic epithelium was first introduced by Robert Meyer in 1924 [53]. According to this theory, multipotent mesenchymal stem cells, originating either from bone marrow or from a specific endometrial niche, are capable of reprogramming. Through this process, they may differentiate into endometrial epithelial and stromal cells at ectopic sites [54]. Exposure to endocrine disruptors and immune dysregulation may facilitate this transdifferentiation [55]. This explanation is frequently invoked to explain unusual cases where endometriosis develops outside the pelvic cavity [56]. In 1927, Sampson, drawing on clinical and anatomical observations, introduced the theory of retrograde menstruation. This concept is still regarded as the leading explanation for most forms of endometriosis [57]. It proposes that viable endometrial fragments reflux through the fallopian tubes into the peritoneal cavity during menstruation, where they adhere, invade, and proliferate on peritoneal surfaces [58,59]. Sampson noted that retrograde menstruation does not adequately explain the occurrence of extraperitoneal lesions or the variability of clinical symptoms arising beyond the pelvis [60]. He was the first to propose hematogenous or lymphatic dissemination of endometrial-like tissue as an alternative explanation [61]. The hypothesis assumes that, at menstruation, endometrial cells infiltrate the uterine vasculature or lymphatics and are transported to distant ectopic sites [62] (Figure 2). These theories collectively indicate that endometriosis results from ectopic implantation or metaplasia of endometrial cells combined with impaired immune surveillance, leading to persistent ectopic growth and inflammation.

### 2.2. Hormonal and Inflammatory Mechanisms

Peritoneal endometriosis represents a chronic inflammatory condition involving enhanced recruitment of peritoneal macrophages and elevated secretion of their products [63]. It is believed that the inflammatory state and immune dysfunction in the peritoneum are both causes and consequences of endometriosis [64]. Accumulating data indicate that hormonal and immune components establish a pro-inflammatory microenvironment, making it easier for endometriosis to persist [65]. Moreover, an immunologically permissive microenvironment within the peritoneal cavity has been implicated in the pathogenesis of endometriosis [66]. Macrophages in the peritoneal cavity of affected women display impaired phagocytic function, while endometriotic cells evade immune clearance by resisting natural killer (NK) cell-mediated cytotoxicity [67,68]. The normal endometrium exhibits cell-specific and menstrual cycle-dependent expression of receptors for estrogens (ERα, ERβ, GPER1), androgens (AR), progestins (PRA, PRB), and glucocorticoids (GR, MR) [69]. Within endometrial stromal cells, progesterone controls retinoic acid production as well as its downstream actions [70]. In addition to signaling via multiple nuclear receptors, retinoids are implicated in regulating normal endometrial physiology and in endometriosis [71]. Dysregulated expression of progesterone receptors, including altered epigenetic programming of the PR promoter, has been observed in the endometrium and in lesions from women with endometriosis [72]. As a consequence, stromal cells exhibit reduced responsiveness to progesterone, a phenomenon commonly referred to as progesterone resistance [73]. Several mechanisms have been implicated in the development of this resistance, including abnormal PGR signaling, chronic inflammation, aberrant gene expression, epigenetic alterations, and exposure to environmental toxins [74]. Although progestins normally reduce cellular proliferation, inflammatory activity, neovascularization, and neurogenesis, these actions are markedly attenuated in endometriosis as a result of progesterone resistance [75] (Figure 2). These alterations highlight the interplay between hormonal imbalance and immune dysfunction, which together create a self-sustaining inflammatory microenvironment that may predispose to malignant transformation. 

Moreover, the chronic estrogen-driven inflammation characteristic of endometriosis results in oxidative DNA damage and impaired cellular homeostasis, establishing a permissive background for oncogenic mutations and neoplastic progression.

### 2.3. Role of Estrogen, Prostaglandins, and Cytokines

Estrogens, classified as steroid hormones, are produced chiefly by the ovaries and the placenta in females [76]. As modulators, estrogens are critically involved in regulating physiological as well as pathophysiological processes [77]. Their biological effects are mediated through estrogen receptors (ERs), primarily ERα and ERβ [78]. Endometriotic tissue exhibits increased expression of steroidogenic enzymes, allowing local, de novo estradiol synthesis independent of ovarian production [79]. Differences in mRNA and protein levels of ERs have been reported between normal endometrium and ectopic or eutopic lesions, with endometriotic stromal cells exhibiting markedly increased ERβ and reduced ERα expression [80]. Estrogen receptor beta (ERβ) contributes to the pathophysiology of endometriosis through multiple mechanisms, including the inhibition of TNFα-induced apoptosis, induction of interleukin-1 expression, and co-stimulation of Ras-related and estrogen-regulated growth inhibitor (REGE) expression in concert with prostaglandin E2 (PGE2) under the influence of estradiol [81]. Upregulation of ERβ is also considered a hallmark of the altered microenvironment of lesions, thereby enhancing the impact of estrogens on inflammation, angiogenesis, and pain pathways [82]. Estradiol acting through ERβ contributes to inflammation by inducing the release of cytokines and prostaglandins from peritoneal macrophages [83,84]. In contrast, ERα displays a dual function, exerting both anti-inflammatory and pro-inflammatory effects [85] (Figure 2). These hormonal interactions emphasize the estrogen-driven inflammatory loop that sustains lesion survival and may contribute to the oncogenic potential of endometriosis.

### 2.4. Immune Dysregulation and Angiogenesis

In women with endometriosis, peritoneal fluid contains elevated numbers of immune cells with heightened susceptibility to apoptosis [86]. It is often characterized by elevated levels of pro-inflammatory mediators such as TNF-α, IL-1β, and IL-6 [87]. Significantly elevated levels of VEGF-A, a key angiogenic factor, have been reported as well [88]. Developing endometriotic lesions stimulate angiogenesis and nerve development and secrete chemoattractant molecules recruiting large numbers of macrophages and NK cells [84]. In addition, patients exhibit dysfunctional macrophages, reduced killing capacity of NK cells, and increased accumulation of regulatory T suppressor cells. Together, these alterations may favor chronic inflammation and contribute to the initiation and progression of endometriosis-associated ovarian carcinoma [89]. These changes may disrupt the surveillance, recognition, and elimination of misplaced endometrial cells, leading to impaired immune responses in the peritoneal cavity [90]. Although retrograde menstruation is common among women, endometriosis is thought to arise in those with impaired innate and adaptive immune responses that cannot adequately eliminate refluxed endometrial tissue [91] (Figure 2). Defective immune surveillance not only facilitates ectopic implantation but also allows accumulation of genetically unstable cells within a pro-angiogenic and pro-inflammatory niche, fostering conditions conducive to malignant transformation.

### 2.5. Genetic and Epigenetic Alterations

Although the etiology of endometriosis remains incompletely understood, the condition demonstrates an estimated heritability of roughly 50%, with approximately 26% attributable to common genetic variation [92]. In the 1980s, the first genetic investigations were conducted in patients with a confirmed diagnosis of endometriosis [93]. Over the years, the relationship between endometriosis and heredity has been demonstrated by several authors through family aggregation studies. Evidence suggests that the accumulation of specific characteristics within a given family cannot be attributed to coincidence [94]. Simpson et al. reported that the risk of developing endometriosis is 8% for daughters of affected mothers and 6% for women with an affected sister [95]. Research has demonstrated that concordance for endometriosis is greater among monozygotic twins compared with dizygotic twins [96]. In addition, a positive family history, particularly with multiple affected relatives, is associated with a higher likelihood of severe disease manifestations [97]. To date, numerous deregulated genes have been identified in endometriotic cells. These genes are involved in diverse biological processes, including apoptosis, vascularization, cell cycle regulation, DNA repair, detoxification enzyme activity, immune system regulation, and cell adhesion [98]. The link between endometriosis and cancer, which may result from genetic mutations, has prompted research into pathogenetic mutations arising in cells during disease development [99]. Exome sequencing revealed that 79% of lesions (EC and/or DIE) carried mutations, with 26% involving *ARID1A*, *PIK3CA*, *KRAS*, or *PPP2R1A*, among which *PIK3CA* and *KRAS* are frequently altered in ovarian cancer [100]. Epigenetic changes represent a common denominator for hormonal, immunological, and inflammatory abnormalities that play a key role in the etiopathogenesis of endometriosis [101]. Environmental factors have the ability to influence the epigenome, which includes DNA methylation and histone modifications [102]. DNA methylation, mediated by DNA methyltransferases, represents a major epigenetic mechanism regulating gene expression [103]. Dysregulation of this process contributes to the abnormal epigenetic profile and progesterone resistance observed in endometriosis [104]. The pathophysiology of endometriosis thus reflects a complex interplay between hormonal signaling, chronic inflammation, immune dysfunction, angiogenesis, and genetic as well as epigenetic abnormalities. Together, these mechanisms not only sustain disease progression but may also facilitate malignant transformation of endometriotic lesions (Figure 2).

### 2.6. The Relationship Between Vaginal Dysbiosis and Endometriosis and Related Cancers

Recent research has revealed that the vaginal and endometrial microbiome plays an important role in maintaining local immune homeostasis within the female reproductive tract [105,106]. In healthy women, the vaginal microbiota is dominated by Lactobacillus species, which produce lactic acid, hydrogen peroxide, and bacteriocins that maintain a low pH and inhibit the growth of pathogenic microorganisms [107].

In contrast, women with endometriosis frequently show a state of vaginal and endometrial dysbiosis, characterized by reduced Lactobacillus abundance and increased colonization by opportunistic species such as Gardnerella, Atopobium, Prevotella, Streptococcus, and *Escherichia coli* [108,109]. These microbial alterations are associated with enhanced secretion of pro-inflammatory cytokines (IL-1β, IL-6, TNF-α) and increased local oxidative stress, which can disrupt the epithelial barrier and promote chronic inflammation in the uterus and peritoneal cavity [110].

Microbiome dysbiosis may therefore serve as both a trigger and amplifier of the inflammatory microenvironment characteristic of endometriosis [111]. The presence of bacterial lipopolysaccharides (LPS) in peritoneal fluid from affected women has been shown to activate Toll-like receptor 4 (TLR4) signaling, stimulating macrophages and enhancing prostaglandin and cytokine release [112]. This “bacterial contamination hypothesis” provides a mechanistic link between dysbiosis, inflammation, and endometriotic lesion persistence [113].

Furthermore, chronic inflammation and oxidative stress induced by dysbiosis can promote DNA damage and epigenetic modifications, thereby potentially contributing to malignant transformation [114]. A similar pattern of microbial imbalance, namely decreased Lactobacillus and increased anaerobes, has been observed in endometrial and ovarian cancers. In these malignancies, dysbiosis may facilitate carcinogenesis by altering local metabolism, modulating immune surveillance, and generating reactive oxygen species that damage DNA [115].

Together, these findings suggest that vaginal and endometrial dysbiosis may represent a shared upstream factor linking endometriosis, inflammation, and endometriosis-associated malignancies. Future studies integrating microbiome profiling with immunologic and molecular data could help clarify whether microbial modulation might serve as a preventive or therapeutic strategy in women at risk for malignant transformation of endometriosis.

## 3. Malignant Transformation of Endometriosis

The relationship between endometriosis and cancer has been the subject of research for several years, and various reports support the hypothesis that some gynecological malignancies arise from endometrial lesions [116]. Endometriosis can develop into an atypical form or even undergo malignant transformation in 0.7–2.5% of cases [117]. Sampson’s criteria are commonly used for the diagnosis of malignant endometriosis and have been proposed. Sampson defined three criteria for the diagnosis of endometriosis-related cancer: (1) evidence of endometriosis adjacent to the tumor, (2) the absence of another primary tumor, and (3) histological evidence consistent with endometrial origin [118]. Furthermore, Scott added the transition from endometriosis to the neoplastic epithelium or stromal component to the criteria [119].

Among histological subtypes, endometrioid adenocarcinoma and clear cell carcinoma are most frequently associated with malignant endometriosis [120]. Endometriosis can be considered a precursor of this tumor, as it is identified in more than 50% of patients with clear cell carcinoma [121]. Endometrioid ovarian carcinoma (ENOC) and clear cell ovarian carcinoma (CCOC) together account for ~25% of all invasive ovarian cancers [122]. These tumors are believed to arise from the neoplastic progression of ectopic endometrial glands subjected to chronic inflammation and hormonal stimulation.

Several risk factors have been described so far that influence the malignant transformation of endometriosis. A higher risk for ovarian cancer is associated with long-standing endometriosis and cases of endometriosis diagnosed at younger ages [123]. *ARID1A* is a tumor suppressor gene located on chromosome 1, encoding the BAF250a protein, a component of the chromatin remodeling complex. Mutations in *ARID1A* have been observed in the two main neoplastic transformations associated with endometriosis [124]. In addition, several genetic alterations, like loss of heterozygosity, *PTEN*, *PIK3CA*, *KRAS*, and *TP53* mutations have been identified in both endometriosis and endometriosis-associated malignancy [125,126,127]. Risk factors may also be linked to prolonged exposure to estrogens or progesterone [128], including early menarche, late menopause, reduced number or duration of pregnancies, and short breastfeeding periods [129]. HRT may reactivate dormant endometriosis and stimulate malignant transformation in women with a history of endometriosis [130]. In obese women, increased aromatization of androgens in adipose tissue leads to the formation of endogenous estrogen, establishing obesity as another contributing factor [131]. Finally, atypical endometriosis, which is characterized by cytological atypia and architectural proliferation, is considered a precancerous lesion with potential for malignant transformation (Table 1) [132].

## 4. Endometriosis and Ovarian Cancer

Ovarian cancer is the eighth leading cause of cancer death in women worldwide [133]. Factors affecting the female reproductive system include ovarian cancer, which has the highest mortality rate, and endometriosis [134]. The disease affects postmenopausal women, with a peak incidence between 55 and 59 years of age [135]. Unfortunately, this cancer is detected at an advanced stage (above average) [136]. Data from 2022 estimates that 324,603 women worldwide will be diagnosed with ovarian cancer. It is estimated that by 2050, the annual incidence of cancer will reach 500,000 per year [137].

### 4.1. Strong Evidence of Association

There is substantial evidence linking endometriosis to a higher risk of ovarian cancer. Endometriosis is significantly associated with a modest increase in the risk of ovarian cancer, according to epidemiological and clinical research [138]. For certain disease subtypes, this association seems to be very noteworthy. Research on women with a history of endometriosis suggests that the increased risk is primarily seen in invasive low-grade serous, CCOC, and ENOC malignancies [3]. The risk of developing epithelial ovarian cancer is also 1.2–1.8 times higher for women with endometriosis, according to a number of large-scale studies. This link is particularly strong for the endometrioid and clear cell subtypes [139]. Among these, clear cell carcinomas seem to show the strongest link to endometriosis, although some studies propose that this may be influenced by the misclassification of high-grade serous tumors as endometrioid types [140]. Furthermore, a link has been found between endometriosis and low-grade serous tumors, although high-grade serous carcinoma usually exhibits little to no correlation with the condition [141]. Crucially, genetic changes that allow for malignant transformation may already be present in endometriotic lesions that are histologically benign [142]. Several researchers have investigated intermediate lesions, even though the exact precursor lesion to endometriosis-associated ovarian cancer (EAOC) has not yet been definitively identified. These include situations in which endometriosis is located next to an ovarian cancer or has histological traits that are different from those of tissues that are obviously benign or obviously malignant [142]. When taken as a whole, these results provide strong evidence that endometriosis, especially when it is chronic or atypical, should be considered a potential cause of ovarian cancer in addition to a benign gynecological condition [143].

### 4.2. Subtypes of Ovarian Cancer

Ovarian cancers are a diverse group of cancers classified based on their histological type and grade of differentiation [144]. Clear cell carcinoma (CCC), endometrioid carcinoma (EDC), mucinous carcinoma, high-grade serous carcinoma (HGSC), and low-grade serous carcinoma (LGSC) are the five major subtypes of invasive epithelial ovarian cancer (EOC), which accounts for more than 95% of all ovarian cancers [3]. Non-epithelial malignancies, including germ cell tumors, sex cord and stromal tumors, and small cell ovarian carcinomas, account for the remaining approximately 5% [145]. Histotypes of ovarian cancer are considered different diseases based on the cell of origin, molecular alterations, clinical behavior, and management [146]. Type I tumors are generally low-grade and progress slowly [147], while type II tumors, such as HGSC, the most common subtype in this group, are high-grade, aggressive, and frequently harbor *TP53* mutations [148], often diagnosed at an advanced stage with widespread peritoneal involvement [149]. Interestingly, EDC and clear-cell carcinoma have been associated with endometrial hyperplasia and endometriosis, with potential to arise in both the endometrium and the ovaries [150]. Recent research has drawn attention to atypical endometriosis, particularly OMAs, as possible precursors to malignancy due to their abnormal cellular features [151,152]. Gaining a deeper understanding of these distinct oncogenic mechanisms is crucial for improving early diagnosis and tailoring treatment strategies to the specific biology of each tumor.

### 4.3. Shared Molecular Pathways and Mechanisms

Emerging molecular evidence indicates that endometriosis and specific histological subtypes of ovarian cancer, particularly CCOC and EDC, share common pathogenic mechanisms and dysregulated signaling pathways. Compared to normal secretory-type endometrium, CCOC is characterized by upregulation of genes involved in cysteine and glutathione synthesis, as well as downregulation of the iron exporter [153]. Mutations in the *ARID1A* gene are frequently detected in ovarian cancers, notably in 46–57% of CCOC and 30% of EC cases [154]. *ARID1A* mutations are exceedingly rare in the healthy population (<0.1%). They can occur at considerably lower frequencies in most other cancer types. It underscores their particular enrichment in EAOCs and supports a shared molecular pathway between endometriosis and these malignancies [155]. In contrast, these mutations are absent in high-grade serous ovarian carcinomas [156]. *ARID1A* is a component of the SWI/SNF chromatin remodeling complex, which governs chromatin structure and gene accessibility [157]. This complex plays a key role in preserving genomic stability by facilitating DNA repair and regulating DNA damage response pathways [158]. *ARID1A* mutations are found in approximately 10% of all cancer types, including those of the endometrium, bladder, stomach, liver, pancreatobiliary system, some ovarian cancer subtypes, and cancer of unknown primary origin [155]. Loss of *ARID1A* function is considered an early event in the malignant transformation of endometriotic tissue and may occur independently of the atypical endometriosis phenotype [159]. The co-occurrence of *ARID1A* loss and *PIK3CA* activating mutations appears to be necessary for tumor development and is known to promote the onset of CCOC via sustained IL-6 production [160]. Remarkably, the PI3K/AKT pathway is one of the main regulators of cancer cell growth, proliferation, differentiation, motility, survival, and glucose metabolism [161]. This pathway is activated when *PIK3CA* mutations occur in parallel with *ARID1A* mutations [161]. This route has been found to be the most commonly changed signaling pathway in epithelial ovarian cancer [162] and is activated in both endometriosis and CCOC [161]. Furthermore, EAOC and the uterine endometrium have all been linked to dysregulation of mTOR signaling [143]. Carcinogenesis is thought to be driven by inflammation in ovarian cancer, and inflammation itself is one of the hallmarks of endometriosis [163]. Oxidative stress, characterized by an imbalance between reactive oxygen species (ROS) and antioxidant defense mechanisms, is widely recognized to play a role in the pathophysiology of endometriosis, causing widespread inflammation in the peritoneal cavity [164]. DNA methylation is an epigenetic process that controls the expression of microRNAs (miRNAs), and changes in this process determine treatment resistance, metastasis, and the course of ovarian cancer [165]. Understanding these similar molecular pathways may lead to the development of targeted therapies, early detection, and preventative measures for endometriosis and ovarian cancer, as well as valuable insights into the malignant potential of endometriosis.

### 4.4. Mechanisms of Carcinogenesis

The presence of ectopic endometrial tissue outside the uterine cavity is known as endometriosis, and it is typically regarded as a benign condition. Nonetheless, there is growing evidence linking it to the emergence of several ovarian cancers, including EDC and CCOC, which have similar pathogenic pathways to endometriosis [153]. Carcinogenesis in endometriosis is a complex process and involves oxidative stress, chronic inflammation, and genetic changes [124]. Chronic inflammation and oxidative stress are key factors in the initiation of carcinogenesis in endometriosis. Notably, a carcinogenic model centered on iron-induced oxidative stress plays a crucial role in the unique microenvironment of endometriotic lesions [164,166]. Endometrial tissue often contains excess iron, which is a factor intensifying oxidative and cellular damage and inflammation [167]. Excess iron not only promotes the survival and proliferation of ectopic endometrial cells but also has a detrimental effect by promoting the formation of free radicals, which lead to oxidative stress, DNA damage, and ultimately contribute to carcinogenesis [167,168,169]. Endometrial cysts accumulate a thick, black fluid called “chocolate fluid” that contains high concentrations of heme, hemoglobin, and iron [170]. The production of ROS by these substances is linked to both excessive and compromised iron metabolism [171]. These iron-driven ROS damage DNA, making the epithelial cells lining the cysts more vulnerable to malignant transformation [172]. Overproduction of ROS upsets cellular equilibrium by destroying proteins, lipids, and nucleic acids [173]. Physiological levels of ROS control important cellular functions such as protein phosphorylation, activation of transcription factors, apoptosis, immunity, and differentiation [173,174]. In cancer cells originating from endometriotic lesions, elevated ROS levels are linked to enhanced proliferative signaling via the PI3K/Akt pathway, often as a consequence of the tumor suppressor *PTEN* being deactivated [175].

The malignant development of endometriotic lesions is thought to begin with mutations in *ARID1A*, a crucial part of the SWI/SNF chromatin remodeling complex [157]. The transition from atypical endometriosis to CCOC is facilitated by the disruption of chromatin remodeling caused by loss of *ARID1A* function, which results in aberrant transcription of tumor suppressor genes, enhanced cell proliferation, and resistance to apoptosis [176]. The PI3K/AKT/mTOR signaling pathway is constitutively activated when *ARID1A* deletion occurs in conjunction with activating mutations in PIK3CA or inactivation of *PTEN* [177]. By encouraging tumor cell survival, proliferation, angiogenesis, and the release of pro-inflammatory cytokines including IL-6, this pathway plays a significant mechanistic role in both endometriosis-associated ovarian and endometrial malignancies [178]. Furthermore, PIK3K/AKT/mTOR is a prospective therapeutic target due to its pivotal function, which may be addressed with pathway-specific inhibitors [177].

Activating mutations in the *PIK3CA* gene or *PTEN* loss of function are often caused by mutations in the *ARID1A* gene. ARID1A encodes a protein that is part of the SWI/SNF chromatin remodeling complex, leading to activation of the PI3K/AKT/mTOR pathway. This cascade promotes tumor cell survival, proliferation, and the secretion of proinflammatory cytokines such as IL-6 [160,161]. *ARID1A* encodes a protein in the SWI/SNF complex responsible for chromatin remodeling. Loss of *ARID1A* function is considered an early stage of malignant transformation of endometriosis, which can occur in CCOC and EC [157,159]. These genetic alterations synergistically increase tumor cell proliferation and resistance to apoptosis. *CTNNB1* mutations may disrupt the Wnt/β-catenin pathway, which is frequently observed in EC. This results in abnormal expression of β-catenin [179,180], which is crucial for regulating tumor cell adhesion, proliferation, and migration [181]. *KRAS* activation due to mutations leads to activation of the MAPK signaling pathway, which regulates key cellular processes, such as proliferation, cell cycle progression, autophagy, and apoptosis [182]. Inactivating mutations in *ARID1A* and *TP53*, leading to loss of their expression, abolish transcription of their target tumor suppressors, such as *CDKN1A* [172,183,184]. Additionally, microRNA dysregulation—such as overexpression of oncogenic miR-200 family members and downregulation of tumor-suppressive miRNAs like let-7i—affects genes controlling cell cycle, apoptosis, and proliferation, thereby indirectly increasing malignant transformation risk [185].

**Figure 3:**
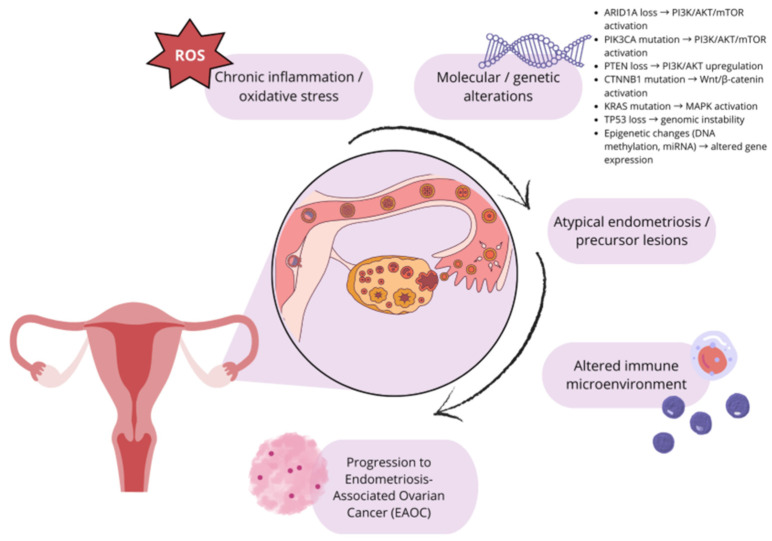
Molecular, inflammatory and immune mechanisms driving the progression of endometriosis to Endometriosis-Associated Ovarian Cancer (EAOC).

The immunological microenvironment within endometriotic lesions is characterized by an infiltration of neutrophils and phagocytic leukocytes [186]. The majority of immune cells in these lesions are macrophages, NK cells, and regulatory T lymphocytes [186]. Endometriosis-driven oxidative stress and immune activation result in the release of inflammatory cytokines, disrupting peritoneal homeostasis and shifting the microenvironment toward a pro-angiogenic state [187]. This promotes the development of a microvascular network and neovascularization of ectopic endometrial tissue [188]. Th17 cells promote angiogenesis in the ectopic endometrium, acting as potent proinflammatory mediators through the secretion of IL-17, which stimulates inflammatory and angiogenic cytokines [189]. All these elements work together to maintain long-term inflammation and cell growth, and create an environment conducive to tumor development. A small percentage of women progress from endometriosis to ovarian cancer, suggesting the involvement of multiple factors. Understanding these complex processes is crucial for strengthening preventive measures and facilitating early detection of endometriosis-associated ovarian cancer (Figure 3).

## 5. Endometriosis and Endometrial Cancer

### 5.1. Summary of Epidemiological Studies

**Figure 4:**
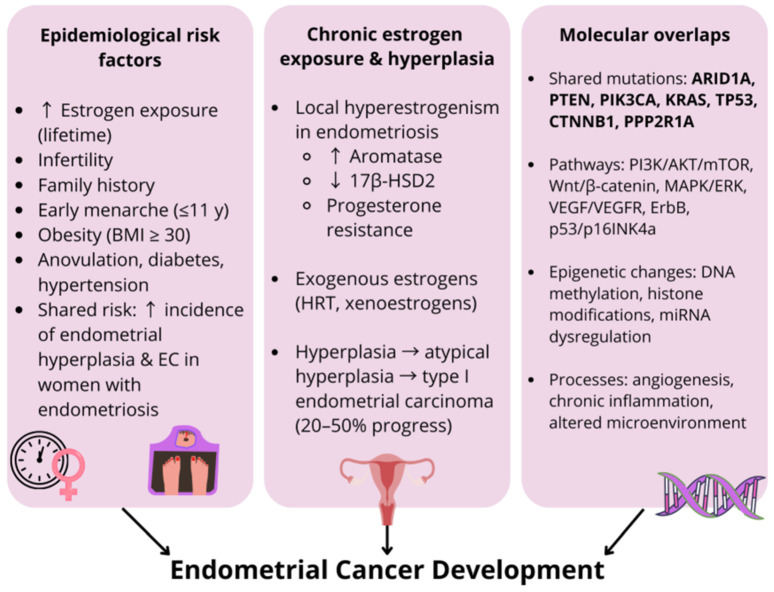
Summary of epidemiological, hormonal and molecular factors linking endometriosis to endometrial cancer development.

Endometrial cancer (EC) is one of the most common gynecological malignancies and represents the most frequently diagnosed gynecological cancer in high-income countries, with its global incidence steadily increasing [190]. It ranks as the seventh-most common cancer among women worldwide [190], with a relatively low prevalence in younger populations; only about 4% of cases are diagnosed in women under the age of 40 [191]. Despite being clinically separate diseases, there is growing evidence that endometriosis and EC may be related epidemiologically. Although the risk is still minimal, women who have endometriosis may be somewhat more likely to acquire EC [192]. However, given that women with endometriosis are more frequently monitored gynecologically, this weak epidemiological association may be partially due to methodological limitations, such as surveillance bias, and differences in endometrial sampling techniques that may mask the actual level of risk [193]. It is interesting to note that infertility and a good family history are two recognized risk factors that both diseases share [194]. These involve overlapping etiological mechanisms, particularly prolonged exposure to estrogens and chronic inflammation [195]. A higher risk of EC has been repeatedly linked to elevated estrogen levels throughout life [196]. Anovulation, diabetes, early menarche (age ≤ 11), obesity (BMI ≥ 30 kg/m^2^), and hypertension are further known risk factors for EC [197]. Although some research indicates that women with endometriosis have higher rates of endometrial hyperplasia and EC, this association is still small and could be muddled by variations in surveillance and diagnosis [198]. The presence of endometriosis does not appear to significantly impact overall survival outcomes in patients diagnosed with EC [198]. Together, the hormonal, histological, and molecular overlaps between EC and endometriosis suggest that there may be shared biological pathways between the two conditions, even if they are not fully captured by the epidemiological evidence currently available. This is true even though population-based data only point to a slight increase in EC risk. Given these epidemiological associations, it is crucial to improve our understanding of the hormonal, histological, and molecular mechanisms potentially linking endometriosis to EC (Figure 4).

### 5.2. Chronic Estrogen Exposure and Hyperplasia

Endometriosis and endometrial carcinoma are gynecological conditions that are dependent on estrogen [199]. The hypothalamic–pituitary–ovarian (HPO) axis modulates cyclical regeneration and differentiation in a healthy endometrium, whereas estrogen and progesterone interact in a closely regulated manner to control the menstrual cycle [200,201]. Endometrial hyperplasia is an example of a hormonal imbalance that can result in disordered endometrial gland proliferation when estrogen stimulation takes place without sufficient progesterone opposition. Such hyperplastic alterations can develop into EC if treatment is not received [202]. Aromatase production in ectopic endometrial tissue is one of the processes that causes a condition of local hyperestrogenism in endometriosis [203]. When aromatase is present and 17β-HSD type 2 is absent, it causes excess estrogen to accumulate in endometriotic lesions [74]. Aromatase is the enzyme responsible for the conversion of androgens to estrogens, including 17β-estradiol [204]. Furthermore, progesterone resistance has been found in endometriotic lesions and eutopic endometrium in women with endometriosis [74]. One of the most important risk factors for endometrial hyperplasia and, in turn, type I EC, which accounts for about 80% of cases [205], is chronic exposure to unopposed estrogen, whether endogenous (for example, through obesity, anovulatory cycles, or estrogen-secreting tumors) [202,206] or exogenous (for example, unopposed estrogen therapy) [207]. Xenoestrogens are synthetic environmental compounds that mimic natural estrogens by binding to ERs and may also contribute to sustained estrogenic stimulation [208]. Although endometrial hyperplasia is not itself malignant, forms containing atypical cells are considered precancerous, with around 20–50% of untreated atypical hyperplasia cases progressing to type I endometrioid adenocarcinoma [209]. These parallels suggest that the hormonal disturbances characteristic of endometriosis could serve as a potential link to premalignant or malignant transformation of the endometrium, with hyperestrogenism as a central, though not solitary, factor (Figure 4) [202].

### 5.3. Possible Molecular Overlaps

Recent studies have shown that the risks of endometrial hyperplasia and EC are significantly higher in women with endometriosis compared to those without [210]. Of particular importance are somatic mutations that are frequently detected in both conditions, especially in genes regulating the cell cycle and tumor suppression. Among the most commonly reported mutated genes in endometriosis are *TP53, PTEN, ARID1A*, *PIK3CA*, *KRAS*, and *PPP2R1A* [211]. Notably, *PTEN* mutations are more prevalent in low-grade endometrioid endometrial carcinoma than in ovarian carcinomas associated with endometriosis [212]. Mutations in *CTNNB1* may define a subgroup of EC patients with a generally favorable prognosis, yet an elevated risk of recurrence [213]. Additionally, discrepancies between *ARID1A* mutation status and its protein expression have been reported in endometrioid endometrial tumors [214]. EC remains one of the few malignancies with increasing incidence and mortality since the mid-2000s [214]. Emerging evidence suggests that *ARID1A* mutation may represent an early event in the malignant transformation of endometrial glandular epithelium, and loss of *ARID1A* expression has also been observed in areas of atypical endometriosis [215]. EC remains one of the few malignancies with increasing incidence and mortality since the mid-2000s [214]. Emerging evidence suggests that *ARID1A* mutation may represent an early event in the malignant transformation of endometrial glandular epithelium, and loss of *ARID1A* expression has also been observed in areas of atypical endometriosis [215]. For this reason, *ARID1A* is regarded as a tumor suppressor gene in endometrial and ovarian malignancies [216]. EC has been found to have a high incidence of *PIK3CA* mutations, up to 50% [217], and these mutations are linked to worse survival outcomes, even in individuals with low-grade tumors [218]. Additionally, *KRAS* mutations are detected in 10–30% of samples of endometrial hyperplasia [219] and in about 16% of instances of EC [220]. Their occurrence in both malignant and non-malignant endometrial tissues suggests potential utility as early molecular indicators of malignancy risk [221]. Similar genetic alterations are often found in endometrioid endometrial carcinoma, suggesting that similar molecular pathways may drive tumor development in both premalignant and malignant lesions [222]. Rather than reflecting actual biological variation, reported frequencies of *KRAS* mutations vary amongst studies, most likely due to methodological variations, such as sequencing depth, tissue sampling, mutation calling criteria, and cohort characteristics [223]. Numerous genetic and signaling pathway changes, such as those involving the Wnt/β-catenin cascade (with APC/β-catenin signaling), PI3K/AKT/mTOR, MAPK/ERK, ErbB, VEGF/VEGFR ligand–receptor, and the p53-P16INK4a pathways, have been linked to the etiology and development of EC [224]. The Wnt/β-catenin pathway is regulated by hormones: progesterone suppresses Wnt/β-catenin signaling during the secretory phase of the menstrual cycle, whereas estrogen activates the pathway during the proliferative phase, increasing β-catenin accumulation in the endometrium [225,226]. Acetylation and methylation are important histone changes that contribute to differential gene expression in endometriotic tissues at the epigenetic level [227]. For example, a decrease in miR-137 expression has been linked to hypermethylation of miR-137 in both serous and endometrioid endometrial malignancies (*p* < 0.01) [228]. In some cases, endometriosis is associated with molecular and microenvironmental changes that may promote malignant transformation, including increased angiogenesis, mutations in cancer-related genes, and chronic inflammation [229]. *ARID1A* mutations, along with PI3K/Akt and mTOR pathway disturbances and epigenetic changes, may contribute to the malignant transformation of endometriosis [161]. Although such genetic changes alone are probably not enough to start tumorigenesis, the presence of somatic mutations in oncogenes like *ARID1A* and *PIK3CA* in both ovarian endometriosis and histologically normal endometrial tissue supports the idea of an underlying molecular predisposition to neoplastic transformation [230]. To better understand the clinical consequences of these findings and determine whether molecular profiling might be used to identify endometriosis patients who are more likely to develop EC, further study is necessary (Figure 4).

## 6. Endometriosis and Cervical Cancer

The association between endometriosis and cervical cancer remains poorly understood and characterized by limited and often contradictory evidence [231]. The strongest correlation was observed in a study that found a significantly lower risk of cervical cancer in women with endometriosis compared with women without endometriosis, suggesting a protective potential for this condition [193]. Increased local immune surveillance and endometriosis-related hormonal changes may be linked to this protective effect [193]. Several theories have been put forth, despite the fact that the mechanisms underlying this possible protective effect are unclear. Local immune surveillance that promotes the elimination of precancerous cervical cells may be a component of chronic inflammation in endometriosis [84,91]. On the other hand, endometriosis’s hormonal environment, including changes in estrogen and progesterone signaling, may lessen cervical epithelial cells’ vulnerability to cancerous transformation [82]. These theories, however, are still theoretical and need more research. Cervical cancer is a cancer strongly associated with chronic infection with high-risk HPV genotypes [232]. Key risk factors for this cancer also include unsafe sexual practices, smoking, lack of HPV vaccination, and lack of regular screening tests, such as Pap smears [233]. A combination of genetic, environmental, and lifestyle factors contributes to its development [232]. One study found no statistically significant association between endometriosis and cervical cancer [234]. Cervical endometriosis itself is rare, but clinical data suggest it may coexist with precancerous conditions and malignant gynecological conditions. For example, among 27 patients with cervical endometriosis, two cases of cervical cancer (7.4%) were diagnosed [235]. Furthermore, one study found that cervical endometriosis coexisted with cervical intraepithelial neoplasia (CIN) in 70% of patients [235]. Although these findings may indicate a possible association, there is currently no evidence for a direct causal relationship between endometriosis and cervical neoplasia [235]. When endometriosis and cervical cancer coexist, it can present diagnostic and treatment difficulties. DIE can mirror the local invasion observed in cervical malignancies, making differentiated diagnosis more difficult, particularly when it affects organs including the uterosacral ligaments, parametrium, rectovaginal space, rectal wall, or bladder [236]. Histopathologically, cervical endometriosis is frequently discovered by accident or after the fact [237]. A number of chronic symptoms, such as heavy menstruation and even potentially fatal bleeding, may be present, or it may stay asymptomatic [237]. At the molecular level, endometriosis is linked to a persistently inflammatory microenvironment that is marked by aberrant expression of estrogen and progesterone receptors, as well as high levels of proinflammatory cytokines such as prostaglandins and IL-6 [238]. Theoretically, these biological variables may aid in the development of cancer in nearby tissues, such as the cervix. Additionally, it is known that oxidative stress and chronic inflammation interact through intricate molecular networks, which may have an impact on the development, progression, and resistance to treatment of tumors [239]. One study indicated that women with endometriosis had a 2.31-fold greater risk of cervical cancer than women without the condition, despite the fact that they did not have a higher frequency of HPV infection [240]. It is important to distinguish between a true biological protective effect and an apparent reduction in risk due to more frequent medical surveillance. An alternative explanation in surveillance bias. Women with endometriosis often have more frequent gynecological visits and routine examinations, including Pap smears [233]. This increased monitoring may lead to earlier detection and removal of precancerous lesions, thereby lowering the observed incidence of cervical cancer compared with the general population, even if the biological risk is not reduced [240].

## 7. Diagnostic and Clinical Implications

### 7.1. Importance of Differential Diagnosis

Distinguishing between endometriosis and gynecologic malignancies is essential because of their similar clinical, radiologic, and possibly even histological features. Endometriosis has been connected to ovarian malignancies, such as endometrioid and clear cell adenocarcinomas, even though it is thought to be a benign condition [241]. Some individuals may experience symptoms mimicking those caused by malignant tumors due to the progressive nature of the disease and involvement of surrounding organs [236,241]. The coexistence of EC or ovarian cancer associated with endometriosis creates additional diagnostic challenges. In such circumstances, determining whether the tumors are synchronous malignancies or metastases from a single site may be nearly impossible [242,243]. When both tissues are subjected to the same carcinogenic stimuli, this complexity increases even more, and it may result in the independent formation of several primary tumors [244]. Misdiagnosis or delayed recognition can result in suboptimal treatment decisions, including undertreatment of malignancy or overly aggressive interventions for benign disease [245,246]. Understanding the molecular overlaps between endometriotic lesions and gynecologic malignancies, such as shared mutations or dysregulated miRNAs, may help identify patients at higher risk for malignant transformation, guiding more intensive surveillance and personalized management strategies. Therefore, a comprehensive diagnostic approach is essential to ensure appropriate therapeutic strategies and improve patient outcomes.

### 7.2. Challenges in Early Detection

Endometriosis represents a significant medical, public health, and socioeconomic burden, as it is a chronic, estrogen-dependent condition that substantially affects patients’ quality of life and reproductive health [247]. Early detection remains a major clinical challenge due to the non-specific nature of symptoms, which frequently overlap with those of gynecologic malignancies [245,246,247,248]. The symptomatology of endometriosis varies depending on the location and extent of ectopic endometrial tissue. Because the lesions respond to hormonal stimulation, many symptoms are cyclical, including dysmenorrhea, menorrhagia, or hypermenorrhea. Other symptoms, such as dyspareunia, dysuria, and dyschezia, are more closely associated with deep infiltrating endometriosis or involvement of the urinary or GI tract [249]. Studies report that pain is the most common symptom of endometriosis (43.5%), followed by palpable findings (28%) [250]. Ultrasonography (USG) is often the first diagnostic method physicians use when patients present with a palpable abdominal lump or mass. Unfortunately, this may turn out to be a malignant transformation of endometriosis of the abdominal wall [251]. Such findings can also occur in various gynecological cancers, but they are not pathognomonic. Similarly, early-stage gynecological cancers often have overlapping or ambiguous symptoms. The most common symptoms in women with ovarian cancer include increased abdominal girth or fullness (26%), abdominal or pelvic discomfort (31%), and GI problems such as bloating and early satiety [252,253]. Abnormal uterine bleeding, pelvic pain, and uterine enlargement are typical symptoms of endometrioid EC [254]. Additionally, advanced EC may present with symptoms such as pyometra or purulent discharge in some patients [255]. In the case of cervical cancer, warning signs include irregular vaginal bleeding, abnormal vaginal discharge, pelvic and abdominal pain, pain during urination, and general symptoms [256,257]. The diagnostic process is further complicated by the fact that DIE lesions or ovarian endometriosis may resemble malignant tumors on imaging studies [258,259]. Occasionally, ovarian endometriosis can undergo malignant transformation, most commonly into endometrioid or clear cell carcinoma, further complicating the situation, making early diagnosis difficult [260]. The lack of reliable and noninvasive diagnostic tools for both endometriosis and early-stage malignancies continues to hinder timely diagnosis. These overlapping clinical and imaging features underscore the need for improved early detection strategies and comprehensive differential diagnosis protocols.

### 7.3. Role of Imaging, Biomarkers, and Surgical Pathology

#### 7.3.1. Imaging Techniques

Imaging plays a key role in the evaluation of lesions potentially associated with gynecological malignancies. Pelvic USG has gained importance in recent years in the diagnosis and staging of gynecological malignancies. Currently, USG is used as a first-line imaging method in combination with pelvic magnetic resonance imaging [261]. Ovarian endometriosis usually presents as cysts with minimal internal echogenicity and is therefore imaged with USG [262]. Furthermore, it allows for determining the extent of GI involvement [262]. However, the US is operator-dependent, requiring a high level of skill and experience [262]. The diagnostic accuracy of pelvic USG has significantly improved, and it is now widely accepted as a first-line method for the evaluation of suspected endometrial and malignant lesions, despite significant interobserver variability [263]. Transvaginal ultrasonography (TVS) is used to identify superficial and DIE. Due to its low cost, wide availability, and high overall diagnostic accuracy, it is considered a first-line diagnostic method [264]. In the evaluation of abnormalities involving the bowel, rectovaginal septum, and other pelvic structures, TVS is diagnostically comparable to Magnetic Resonance Imaging (MRI) when performed by a physician experienced in ultrasound imaging [264,265].

MRI provides improved soft tissue contrast, which is particularly useful in unclear or complex cases [266]. In EC, MRI reliably assesses uterine and cervical myometrial invasion, as well as ectopic growth, which is crucial for surgical planning [267]. In cervical cancer, MRI can differentiate between early and advanced disease, supporting appropriate treatment stratification between surgery and chemoradiotherapy [267]. MRI is still recommended to distinguish benign from malignant ovarian lesions [262]. Moreover, it aids in the diagnosis and staging of EAOC by identifying specific morphological and signal characteristics [268], such as unilocular cystic lesions with hypointense signals on T2-weighted images [269]. However, MRI can help distinguish EAOC from non-EAOC but does not allow for a definitive histological classification [262].

CT is crucial for identifying lymph node involvement, distant metastases, and extrauterine spread [270]. According to some studies, CT procedures may be more useful in the noninvasive evaluation of endometriosis if appropriately timed to the menstrual cycle [271]. However, especially in situations that are unclear or borderline, CT by itself is unable to accurately distinguish between benign endometriotic lesions and cancers [271].

Therefore, for lesions suspected of malignancy, a comprehensive imaging approach is often necessary to establish an accurate diagnosis and guide effective treatment planning. Furthermore, ongoing developments such as AI-based image analysis and standardization of imaging protocols should help overcome current obstacles, reduce diagnostic variability, and improve accuracy in the near future.

#### 7.3.2. Biomarkers

Biomarkers are often used to aid in the diagnosis of gynecological and oncological conditions, but their ability to differentiate endometriosis from gynecological malignancies remains limited due to their low specificity [272]. In Table 2, there are presented biomarkers in diagnosis of different gynecological abnormalities. CA-125, although commonly elevated in endometriosis [273], is also elevated in various conditions, such as ovarian cancer, pelvic inflammatory disease, and even during menstruation, reducing its diagnostic specificity [272,273,274]. Studies have shown that CA-125 levels are significantly higher in patients with endometriosis compared to controls throughout the menstrual cycle [274]. Extremely elevated levels of some tumor markers can be observed in benign conditions such as endometriosis [274]. Tumor markers such as CA19-9, carcinoembryonic antigen (CEA), sialyl-Lewis X (SLX), and lactate dehydrogenase (LDH) showed a difference in their concentrations in patients with ovarian cancer vs. patients with endometriosis [275]. HE4 is a much more specific marker than CA-125 (93% vs. 78%) for ovarian cancer and is usually not elevated in benign conditions, including endometriosis [276,277]. The Risk of Ovarian Malignancy Algorithm (ROMA) classifies individuals into high- and low-risk groups for ovarian cancer based on factors such as menopausal status, HE4, and CA-125 values [276]. Notably, a promising noninvasive method for identifying and tracking ovarian cancer is urinary HE4 testing [277].

Researchers are looking into new biomarkers in addition to traditional ones. Numerous proteins, such as serum paraoxonase/arylesterase 1, hemoglobin subunit beta, selenoprotein P, and complement component C9, have been reported to have a favorable correlation with endometriosis. These findings may be due to underlying mechanisms such as metabolic imbalance, oxidative stress, and immune dysregulation [272]. The significance of circulating non-coding RNAs for diagnosis is becoming more and more apparent. The deregulation of certain miRNAs in endometriosis and ovarian cancer has been implicated in a possible role in early neoplastic transformation, particularly in EAOC [278]. Members of the miR-200 family, as well as other miRNAs such as miR-21, miR-200, and miR-145, have demonstrated promise in distinguishing malignant from benign gynecologic conditions, though further clinical validation is necessary [278]. Differentially expressed miRNAs may also contribute to the pathogenesis of endometriosis itself [279], and tailored miRNA panels are being investigated for their potential use in the early detection and management of ovarian cancer depending on disease stage [280]. In contrast to CK-19, which shows no discernible diagnostic value, promising options include non-neuronal enolase, vitamin D-binding protein, and urine peptide profiles, which have demonstrated an improved capacity to distinguish between women with and without endometriosis (Table 3) [281]. Knowledge of molecular alterations in endometriosis and early-stage ovarian cancers, including miRNA expression profiles, could facilitate biomarker-based screening and allow for the personalization of hormonal or targeted therapies in high-risk individuals.

**Table 2:** Selected Biomarkers in the Diagnosis of Endometriosis and Gynecologic Malignancies.

Biomarker	Associated Condition(s)	Clinical Role	Specifity/Sensitivity	Remarks
CA-125	Endometriosis, ovarian cancer	Diagnostic	Low specificity in endometriosis; ~78% specificity in ovarian cancer [277]	Elevated in menstruation, PID, pregnancy, and malignancies; limited diagnostic precision [274,275]
HE4	Ovarian cancer	Diagnosis and monitoring	~93% specificity [277]	Rarely elevated in benign conditions; detectable in urine [278]
ROMA (CA-125 + HE4 + menopausal status)	Ovarian cancer	Risk stratification (low/high)	Higher accuracy than single markers [277]	Used to evaluate adnexal masses
CA19-9	Endometriosis, EAOC	Potential differential marker	No precise sensitivity data	Significantly different levels in EAOC vs. endometrioma [273]
CEA	EAOC	Tumor marker	No precise data	Often elevated in GI cancers, but also in EAOC [273]
SLX (Sialyl Lewis X)	EAOC	Supportive biomarker	No precise data	Shows differences between EAOC and endometrioma [273]
LDH	EAOC	Metabolic marker	No precise data	May indicate malignant transformation [273]
miR-200 family	Endometriosis, ovarian cancer	Differential diagnosis	Promising, needs clinical validation [281]	Involved in epithelial–mesenchymal transition (EMT) and tumor progression
miR-21, miR-145	Endometriosis, EAOC	Molecular biomarkers	Dysregulated in both conditions [281]	May be involved in early neoplastic transformation
C9, Selenoprotein P, PON1, HBB	Endometriosis	Experimental biomarkers	No precise data	Reflect immune activation, oxidative stress, and metabolic alterations [274]
NSE (non-neuronal enolase)	Endometriosis	Differential diagnosis	No precise data	More effective than cytokeratin 19 in distinguishing endometriosis [272]
Vitamin D binding protein	Endometriosis	Diagnostic aid	No precise data	Promising urinary biomarker [272]
Cytokeratin 19	Endometriosis	-	Not diagnostically relevant [272]	No significant difference between affected and unaffected women

**Table 3:** A summary and comparison of risk measure, type of studies included in the research, key findings and evidence quality in different types of cancers.

Cancer Type	Risk Measure	Study Type	Key Findings	Evidence Quality
Ovarian cancer	1.2–1.8 times higher risk ratio in the group of patients with endometriosis [139]	Systemic review, Narrative review,	Corelation with the mutations in ARID1A gene and PIK3CA activating mutations, PTEN loss, CTNNB1 mutations, KRAS mutations, TP53 loss [154,160,181,182], ARID1A mutations occur in 46–57% of CCOS [154], ARID1A mutations are absent in high-grade serous ovarian carcinomas [156]	High
Endometrial cancer	Risk may be higher due to elevated estrogen levels in group of women with endometriosis [197]	Systemic review, Meta-analysis, Literature review	PTEN, TP53, ARID1A, KRAS, PIK3CA and PPP2R1A are common mutations overlap between endometriosis and EC [211], Key pathways leading to EC development include Wnt/β-catenin, MAPK/ERK, PI3K/AKT/mTOR, VEGF/VEGFR and p53-p16INK4a [224]	Moderate
Cervical cancer	Endometriosis coexisted with cervical intraepithelial neoplasia (CIN) in 70% of patients [235]	Systemic review, Meta-analysis, Case report, Literature review	CA-125 is often elevated in endometriosis but also can be in other conditions decreasing its diagnostic value [274], CA 19-9, CEA, SLX and LDH present differing levels in endometriosis and ovarian cancer [276,277], HE4 is more specific comparing to CA-125, however is commonly not elevated in benign lesions [277]	Low

#### 7.3.3. Surgical Pathology

When imaging studies and biomarkers do not yield a definitive diagnosis, surgical intervention, most often laparoscopy, may be considered, particularly in patients with inconclusive imaging findings or in whom empirical treatment has failed or is not suitable [282]. During laparoscopy, endometriotic lesions can be observed macroscopically as superficial “powder-burn” marks on the ovarian surface or peritoneum, varying in color from white to red, brown, or blue [283]. Lesions may also present as deeper nodules or cysts [283]. While laparoscopy is no longer considered the gold standard for diagnosing endometriosis, it still plays a crucial role in selected cases. Histopathological examination of excised tissue remains essential to confirm endometriosis and to rule out malignancy [282,284]. Notably, EAOCs, especially endometrioid and clear cell carcinomas, can originate within endometriotic foci [285]. Therefore, careful pathological evaluation is particularly important when lesions display atypical characteristics such as rapid growth, solid elements, or resistance to hormonal therapy [249].

## 8. Future Directions and Research Gaps

Despite growing awareness of the association between endometriosis and gynecological malignancies, significant gaps remain in our understanding of its mechanisms and clinical implications. One of the most pressing needs is the implementation of large-scale, prospective cohort studies to more precisely identify risk factors, pathways of malignant transformation, and long-term outcomes for women with endometriosis [286]. The design and implementation of strong clinical trials, which are essential to the creation of successful medical therapies, will also be necessary to advance knowledge in this field [287]. Specifically, the gold standard for assessing the safety and effectiveness of novel therapeutic approaches and contrasting them with current treatments is still randomized, double-blind, case–control trials [288]. The creation of trustworthy, non-invasive diagnostic instruments based on verified biomarkers is equally crucial. Despite being a primary area of research, there is currently not enough data to conclusively confirm their accuracy in identifying pelvic endometriosis or differentiating it from other benign ovarian lesions [289]. However, the Enzian classification highlights that imaging modalities like transvaginal ultrasound or MRI may frequently provide adequate information for preliminary diagnosis and treatment planning, minimizing the need for immediate invasive procedures [282]. For diagnosis, laparoscopy combined with histological confirmation is still the gold standard. To improve predictive models and enhance diagnostic and prognostic capabilities, future research should focus on combining multiomics data such as proteomics, metabolomics, and genomics [289]. Early detection and prognosis of ovarian cancer may be possible through the use of advanced data analysis methods combined with biomarker-based algorithms [289]. Among the most studied possibilities are microRNAs, CA-125, HE4, and other inflammatory or angiogenic markers; however, well-designed trials are still required to show their clinical use [273,276,281]. Personalized medicine is also gaining popularity [290] to tailor prevention and treatment plans to each patient’s profile. Considering risk-based classification and more targeted therapies, a better understanding of genetic variation offers new opportunities for personalized pharmacotherapy [291]. In particular, emerging evidence highlights the therapeutic potential of targeting the PI3K/AKT/mTOR signaling pathway, which is frequently dysregulated in EAOCs [161,224]. Preclinical and early phase clinical studies suggest that inhibitors of this pathway such as everolimus, temisirolimus or alpelisib may suppress tumor growth and overcome resistance to conventional chemotherapy by modulating cell proliferation, apoptosis, and angiogenesis [292,293,294]. Further trials are needed to evaluate their safety and efficacy in the specific context of EAOCs. Moreover, the chronic inflammatory microenvironment and immune dysregulation described in endometriosis may also provide a biological rationale for the application of immunotherapy [295]. Checkpoint inhibitors targeting PD-1/PD-L1 or CTLA-4 pathways have shown promise in several gynecologic malignancies and could be particularly relevant for EAOCs characterized by high levels of immune infiltration and cytokine-mediated signaling [296,297]. Combining immunotherapeutic approaches with molecularly targeted agents might enhance antitumor responses and improve long-term outcomes [296,297]. Furthermore, combining new therapies with established fertility preservation techniques protects hormonal and reproductive health while improving the long-term quality of life in younger cancer patients [298]. Future research should explore how molecular overlaps between endometriosis and gynecologic malignancies can inform clinical management, including surveillance of high-risk patients, personalized hormonal therapy, and biomarker-guided screening programs. Close interdisciplinary collaboration between experts in gynecology, oncology, molecular biology, and epidemiology will be essential to address these complex research gaps and pave the way for more personalized and effective treatment regimens.

## 9. Conclusions

This review highlights the strong association between the presence of endometriosis and malignant transformation. The highest risk of malignant transformation is described for ovarian lesions and the development of ENOC cancer and clear cell carcinoma. A somewhat smaller association has been demonstrated for EC, and contradictory information, sometimes even suggesting a protective potential for endometriosis, has been reported for cervical cancer. Research indicates that malignant transformation is a multifactorial process. It may depend on the mutations, the type of endometrial lesions, hormonal imbalances, and inflammation, which is a multifactorial process. A thorough understanding of the mechanisms of malignant transformation is essential from a clinical perspective for implementing patient-specific treatment. To fully understand this multidisciplinary mechanism, interdisciplinary collaboration and cooperation between gynecologists, oncologists, immunologists, and molecular biologists will be necessary.

## Figures and Tables

**Table 1:** Table comparing benign vs. malignant features of endometriosis-associated lesions.

Feature	Benign Endometriosis	Malignant
Histological architecture	Glands and stroma outside uterus, no invasion [1,2,3,12,13,14]	Loss of architecture, presence of dysplasia and cellular atypia [32,33,34,38,39,40]
Presence of atypia	Absent or minimal [14,15]	Marked atypia and dysplastic changes [32,38,43]
Genetic mutations	Occasional somatic mutations [51]	Frequent mutations in *ARID1A*, PTEN, PIK3CA [34,38,40,92]
Inflammation	Chronic peritoneal inflammation [2,7,51,63,84,91]	Sustained inflammatory signaling, promoting carcinogenesis [67,68,84,91]
Angiogenesis	Increased VEGF levels in peritoneal fluid [20,88]	VEGF-driven neovascularization contributing to tumor progression [38,88]
Estrogen dependence	Estrogen-responsive tissue [48,50]	Estrogen promotes tumor growth via steroidogenic gene activation [80,82]
Progesterone resistance	May be present, variable [50,51]	Pronounced resistance linked to poor therapeutic response [74,75]
Immune dysfunction	Altered macrophage and NK cell activity [50,51]	Immune evasion mechanisms active in tumor microenvironment [67,84,91]
Risk of malignant transformation	Low, estimated ~1–2.5% in specific subtypes [56]	High in long-standing endometriosis with atypia or associated ovarian neoplasms [56,92]
Common associated cancers	None	CCOC, endometrioid carcinoma (EDC) of the ovary and genital tract [56,92]

## Data Availability

No new data were created or analyzed in this study. Data sharing is not applicable to this article.

## Abbreviations

The following abbreviations are used in this manuscript:

CA-125	Cancer Antigen 125
CCOC	Clear Cell Ovarian Carcinoma
CEA	Carcinoembryonic Antigen
CIN	Cervical Intraepithelial Neoplasia
CT	Computed Tomography
DIE	Deep Infiltrating Endometriosis
EAOC	Endometriosis-Associated Ovarian Cancer
EC	Endometrial Cancer
EDC	Endometrioid Carcinoma
ENOC	Endometrioid Ovarian Carcinoma
ERβ	Estrogen Receptor Beta
ERs	Estrogen Receptors
GI	Gastrointestinal
HE4	Human Epididymal Protein 4
HPV	Human Papillomavirus
HRT	Hormone Replacement Therapy
LDH	Lactate Dehydrogenase
miRNAs	microRNAs
MRI	Magnetic Resonance Imaging
NK	Natural Killer
OMA	Ovarian Endometrioma
PGE2	Prostaglandin E2
REGE	Estrogen-Regulated Growth Inhibitor
ROMA	Risk of Ovarian Malignancy Algorithm
ROS	Reactive Oxygen Species
SLX	Sialyl-Lewis X
SUP	Superficial Peritoneal Endometriosis
TVS	Transvaginal Ultrasonography
USG	Ultrasonography
